# “Dare to Share”: Improving Information Sharing and Risk Assessment in Multiteam Systems Managing Offender Probation

**DOI:** 10.3389/fpsyg.2022.869673

**Published:** 2022-07-08

**Authors:** Sara Waring, Ellise Taylor, Susan Giles, Louise Almond, Vincent Gidman

**Affiliations:** ^1^Department of Psychology, University of Liverpool, Liverpool, United Kingdom; ^2^Devon and Cornwall Police, Exeter, United Kingdom

**Keywords:** information sharing, probation, police, offender management, multiteams

## Abstract

Managing offenders released from prison requires a multiteam system (MTS) approach, with teams from across police, probation, and other criminal justice agencies. However, public inquiries highlight current approaches are impaired by poor information sharing that compromises risk assessment and recall decisions, which can allow serious further offences to occur. Little research has focused on the causes of these information sharing difficulties. The current study draws on the perspectives and experiences of probation and police officers to improve understanding of causes of information sharing difficulties. The research is conducted within the context of a new enhanced information sharing ‘Direct Access’ initiative implemented in one region of the UK (Devon and Cornwall) that permits probation to directly access police information technology systems. This provides a novel opportunity to cross validate MTS theory to the real-world context of offender management and considers what works in practise to overcome information sharing challenges. Eleven semi-structured interviews were conducted with police (*N* = 4) and probation (*N* = 7) officers. Thematic analysis revealed six themes: i) information sharing difficulties and impact; ii) causes of information sharing difficulties; iii) impact of ‘Direct Access’ on information sharing practices; iv) workload inequality; v) training; and vi) evolution of ‘Direct Access’. Overall, findings highlight that information sharing difficulties are causes by not knowing what information to request or share, limited resources, lack of clarity about General Data Protection Regulation and concern about consequences of breaching this. These barriers can result in delays and failures to share information, which hinders the accuracy of risk assessments and ability to safeguard. Findings also highlight that providing statutory partners with ‘Direct Access’ to I.T systems can improve the relevance and timeliness of information. However, ‘daring to share’ is not enough to address trust issues without also clarifying expectations regarding information use and perceived workload inequalities.

## Introduction

Multiteam systems (MTS) are teams of teams working toward a shared superordinate goal but with unique subgoals at individual or team levels (Marks et al., 2001, 2005). The flexibility of these structures, specialization of skill sets and ability to pool knowledge and resources, make MTSs ideal for operating in extreme environments characterized by risk and uncertainty, rapidly changing situation, and need for fast response (Shuffler and Carter, 2018). This includes managing offenders released on license from prison to serve a period of court sentence in community settings, which is important for challenging them *“to change their offending lifestyles and to confront difficult issues”* (House of Commons Justice Committee, 2011, p. 5). The MTS responsible for managing offenders on license is comprised of individuals and teams from several departments and agencies across the criminal justice system, working toward the shared superordinate goal of protecting society through preventing crime but with independent and interdependent subgoals at individual, team, and agency level (Brown et al., 2021). For example, the probation service is responsible for supervising offenders on license to ensure they continue to meet probation conditions, whilst police are responsible for preventing, detecting, and investigating crime. Probation relies on police for information about offenders on license to inform risk assessments, such as offense activity, interactions with other known criminals or locations that breach the terms of license. Police relies on probation to effectively monitor, support, and recall offenders to prison if necessary to prevent further offending.

However, with more than 8,000 offenders on license committing further offenses in 2021 alone (Ministry of Justice, 2021), questions are being raised about the effectiveness of multiagency working and risk assessment in probation settings. One of the key problems to be identified within this large and complex MTS is poor information sharing, leaving probation officers with outdated, unreliable, and incomplete information (Fitzgibbon, 2008; Ricks et al., 2016). Poor information sharing compromises the accuracy of risk assessment and decisions regarding whether to recall an offender on license to prison, which can allow serious further offenses to occur (Florence et al., 2014; Matz and Kim, 2017; Brown, 2018), such as the murders of Jessica Chapman, Holly Wells (Bichard, 2004), and Tanis Bhandari (Plymouth Safeguarding Board, 2020). But despite the importance of this issue, little research has focused on understanding the causes of these information sharing difficulties, limiting ability to improve practices within offender probation.

Accordingly, the following study draws on the perspectives and experiences of probation and police officers to improve understanding of causes of information sharing difficulties within the large MTS responsible for managing offender probation and how these may be addressed in practice. We focus on one region of the United Kingdom, Devon, and Cornwall, where a new enhanced information sharing initiative called “Direct Access” has been implemented to support statutory partners without creating excessive and unmanageable demand on limited police resources. What makes this unique is that it is the first time a United Kingdom based police organization have permitted an external agency to directly access its information technology (I.T) systems. This provides a novel opportunity to cross validate MTS theory to the real-world context of offender management and consider what works in practice to overcome information sharing challenges.

### Information Sharing in Offender Probation Settings

All organizations in the United Kingdom, including police and probation services, are subject to the Data Protection Act 2018 and General Data Protection Regulation (GDPR), which controls how personal data is shared and used to ensure fairness, lawfulness, and transparency. Processing of personal data relating specifically to “law enforcement purposes” is covered by the Law Enforcement Directive legislation introduced by the European Union in May 2018 to parallel GDPR. Police and probation have legal powers that allow and require them to share personal data under certain conditions, including the Crime and Disorder Act 1998, the Police and Justice Act 2006, and the Criminal Justice and Court Service Act 2000. In effect, personal data can be shared if it is needed for preventing, detecting, investigating, or prosecuting criminal offenses, or executing criminal penalties and safeguarding against threats to public security, so long as this is proportionate and targeted to a specific need (College of Policing, 2018). Despite this, public inquiries repeatedly highlight difficulties with information sharing and coordination within offender management (Goodwin et al., 2012), and other large and complex MTSs operating in extreme environments (Pollock, 2013). Indeed, a recent United Kingdom safeguarding review into the murder of Tanis Bhandari by an offender on license concluded that information sharing between police and probation services was “*generally ineffective*,” compromising assessment of risk posed (Plymouth Safeguarding Board, 2020). Such issues are by no means new or restricted to United Kingdom criminal justice agencies, with similar problems identified in the United States and other countries (Kelley, 2003; Lennox et al., 2012).

To date, limited research has focused on understanding the underlying causes of information sharing difficulties in probation settings. What evidence does indicate is problems with clarifying whose role and responsibility it is to share information across large government agencies including within criminal justice settings (Kelley, 2003; Gil-Garcia et al., 2019). One factor likely to contribute to this lack of clarity is not having capacity to invest in inter-agency working (Matz and Kim, 2017). Both probation and police have experienced significant funding cuts due to austerity measures, and priority for allocating remaining finite resources is often given to addressing intra-rather than inter-agency goals (Matz and Kim, 2017). Within the MTS literature, evidence also highlights that disparity in membership can affect willingness to invest in inter-agency working, particularly if the objectives of one group receive greater priority over others (Shuffler et al., 2015). This may be the case in probation settings with chief police officers being found to view inter-agency information sharing less favorably than chief probation officers due to the greater resource burden placed on police, whilst probation is perceived to receive the greater benefit (Kim et al., 2016). This suggests that any information sharing strategies implemented would need to reduce resource burden, particularly for police.

Another key problem for large MTSs operating in complex environments, such as probation management, is knowing what information is relevant to share. Typically, probation will notify police about offenders released on license and will then be reliant on police to share what information they have that is relevant for improving assessment of risk posed by each offender. Yet, evidence highlights problems with recognizing what is relevant to share across criminal justice agencies, leading to disparities in what is shared and what is needed (Kelley, 2003). This is a common issue for large MTSs comprised of multiple teams working toward both interrelated and unique goals (Chatzimichailidou et al., 2015; Waring et al., 2019). It is not as simple as being able to just share all information with everyone because vast amounts of information are distributed across large and complex networks, not all of which is relevant to each role. Sharing everything with everyone would be inefficient and cognitively demanding (Stanton et al., 2006). Instead, effective information sharing requires that each MTS member be able to access information of relevance to their role when they need it (Stanton, 2016). However, questions remain as to how best to achieve this within large and complex MTSs operating in extreme environments, including probation.

To date, much of the research into information sharing has focused on traditional single-agency team structures where membership is stable. The findings of such research highlight the importance of developing familiarity, trust (Jarvenpaa and Keating, 2011; Ren and Argote, 2011), and shared knowledge of who knows what over time (transactive memory; Wegner et al., 1985; Heavey and Simsek, 2015) for improving the relevance of information sharing. However, the extent to which these findings translate to large multiagency MTSs operating in extreme environments is questionable (Davison et al., 2012; Shuffler et al., 2015; Shuffler and Carter, 2018; Waring et al., 2018, 2020). In probation settings it would be difficult to develop familiarity, trust, and knowledge of who knows what as membership is unstable across agencies and information needs rapidly change as new offenders are regularly released from prison with different licensing conditions and probation officers supervising them. Information pertaining to offenders on license is also distributed across large networks and systems, making it difficult to identify who needs what and when. Further research focus is needed on information sharing in large MTSs with unstable membership operating in extreme environments to identify practical solutions for improving ability to recognize what information is relevant to share (Shuffler and Carter, 2018; Waring et al., 2018, 2020).

Furthermore, even when agencies correctly identify what information is relevant to share, organizational culture could still create barriers to disclosure. Evidence highlights a culture of secrecy within policing, which produces a mantra of “*need to know*” rather than “*dare to share*,” and lack of clarity around data protection further exacerbates this reluctance to disclose information (Kelley, 2003; Maras, 2017; Gil-Garcia et al., 2019). Lack of trust in how other agencies may use information increases concerns that intelligence will be compromised or lost (Maras, 2017) and investigations hampered by offenders being made aware of police activities (Aden, 2018). Yet, withholding information compromises risk assessments and increases the potential for offenders on license to commit further serious offenses (Maras, 2017). The United Kingdom has sought to address these issues through the introduction of Integrated Offender Management teams (IOMs) to work with offenders in the community (Home Office, 2020). IOMs are comprised of representatives from police, probation, and healthcare, forming a dedicated multiagency team that works with an offender, allowing familiarity and trust to develop between members in this more stable team structure. Whilst there is evidence that IOMs help to reduce offending for those on license (Wong, 2013; Sleath and Brown, 2019), this is a resource intensive approach that is only implemented for the small number of offenders on license viewed to be at very high-risk of causing harm or reoffending (Hadfield et al., 2021). With more than 220,000 offenders typically on probation across a 3-month period^1^, police and probation services do not have the resources to implement IOMs for all offenders on license, nor may such an approach be warranted.

Another less resource intensive option is to develop secure I.T systems that would allow information to be shared and coordinated across criminal justice agencies (Gil-Garcia et al., 2019; Kamau et al., 2021). Advances in technology make it possible to allow information uploaded onto one agency’s system to be shared across other systems, although the financial costs associated with overhauling systems to make them compatible can limit the implementation of this option. However, one advantage is that it would allow information to be accessed by MTS members even when they are geographically dispersed (Sullivan et al., 2015), as has been the case throughout the COVID-19 pandemic. Another is that it would allow MTS members from across teams and agencies to directly access information when needed rather than relying on the capacity of others to share it (Matz and Kim, 2017). Indeed, there is some evidence that this type of approach can improve the timeliness and relevance of information access in criminal justice settings, but such work has focused on intra-agency processes within police rather than across agencies (Kim et al., 2010). Whilst it is technologically possible to develop secure systems for improving access to information, there is a lack of research addressing how best to overcome cultural issues with reluctance to share sensitive information in large MTSs such as those in criminal justice or security settings.

### Current Study

Public inquiries highlight difficulties with information sharing in large MTSs responsible for managing offender probation, compromising risk assessments, and allowing further offending to occur. For example, following the homicide of Tanis Bhandari in 2015 a safeguarding adult review was conducted (Plymouth Safeguarding Board, 2020). The killer was on license at the time of the homicide after being arrested and bailed 2 weeks previously, but police did not inform his probation officer. The review noted that “*there was a chance Mr. Pemberton would not have been at liberty on that night*” had that key license information been available to police, or if details of his arrest were made available earlier to probation staff. However, limited research has focused on the underlying causes of such difficulties and the extent to which findings from traditional stable teams translate to large MTSs with unstable membership operating in extreme environments with changing information needs remains questionable (Davison et al., 2012; Shuffler and Carter, 2018). Even when research has focused on large MTSs in extremis, focus tends to be directed to events of limited duration such as disasters (Marks et al., 2005; Waring et al., 2018, 2020), rather than to contexts that pose ongoing risks to public safety by prolific and violent offenders, making it difficult to identify practical solutions. What evidence does suggest is the need for strategies to minimize resource burden, particularly for agencies with a smaller membership or benefit weighting. Having direct access to information through secure I.T systems may provide a less resource intensive approach but the lack of research focus on how to overcome cultural barriers to “*dare to share*” creates unknowns for implementing such systems in practice.

Accordingly, the following case study focuses on one region of the United Kingdom where Devon and Cornwall Police Force introduced a new enhanced initiative called “Direct Access” in 2018 to support information sharing with the probation service without creating excessive and unmanageable demand on limited police resources. The initiative required the creation of Information Assurance Agreements between police and probation. A new bespoke I.T program was developed, and probation staff were selected, vetted, and trained on the new “Direct Access” system, which allowed them to access daily police reports (Qlikview), police incident logs (Webstorm), and police crime and custody reports (Unify). Physical access to police I.T required an uplift in I.T within probation estates. Where this was not possible, secure police laptops were provided. To allow the “Direct Access” project to evolve, an internet based “secure link” solution was later designed to negate the need for provision of Police I.T. This initiative is unique in that it is the first time a United Kingdom based police organization has permitted an external agency to directly access its I.T systems. Consequently, this provides a novel opportunity to cross validate MTS theory to the real-world context of offender management to consider the underlying causes of information sharing problems and how they can be overcome in large MTSs with unstable membership operating in environments with ongoing risk to public safety and shifting information requirements.

## Methodology

Whilst problems with information sharing across the large and complex MTSs responsible for offender management is by no means unique, the implementation of “Direct Access” represents the first time a United Kingdom police organization has permitted an external agency to directly access its I.T systems. Accordingly, a case study design was used to examine this contemporary phenomenon to understand how the introduction of “Direct Access” has affected information sharing practices between Devon and Cornwall police and probation services (Yin, 2018). A qualitative research approach was used to collect in-depth data that would provide a deeper understanding of this novel phenomenon (Bhandari, 2020). To enhance methodological fit, we adopted an inductive approach, deriving meaning from the data to develop nascent theory rather than taking a deductive approach that would require *a priori* assumptions to be made about data to test hypotheses (Edmondson and McManus, 2007). Data was collected using semi-structured interviews, with a question schedule used to discuss the lived experiences of police and probation staff, but with flexibility to recognizing their expertise and allow them to raise additional topics they perceived to be important (Guest et al., 2013).

### Sampling and Participants

Participants were recruited using a theoretical sampling approach, selecting participants based on a set of specific characteristics to develop and refine theory (Glaser and Strauss, 2017). Selection criteria included being a serving police or probation officer from Devon and Cornwall with lived experience of information sharing processes relating to offender management both prior to and post the implementation of “Direct Access,” thereby allowing them to make direct comparisons. A detective sergeant working in the IOM team identified individuals from police and probation services that met these criteria and acted as a gatekeeper, e-mailing an invitation and information sheet to the pool of potential participants (5 police officers and 9 probation officers). Those that were willing to participate were instructed to contact the research team directly to arrange a suitable time to be interviewed. In line with a theoretical sampling approach, interviews were transcribed and analyzed throughout the recruitment process, with further interviews being scheduled until data saturation was reached (Fusch and Ness, 2015). Qualitative research literature suggests this can occur between six and 12 interviews (Boddy, 2016), and was achieved within 11 interviews in this study.

In total, four police and seven probation officers were interviewed between August and September 2021. Several had been involved in the design and delivery of “Direct Access,” helping to develop a broad understanding of information sharing difficulties, how cultural barriers were being overcome, and the impact of “Direct Access” on information sharing practices in offender management. Police participants included a deputy chief constable with strategic responsibility for overseeing the implementation of “Direct Access,” a sergeant responsible for managing “Direct Access,” and two constables involved with the technical development of “Direct Access.” Probation participants included two senior probation officers, one of whom was involved in the development of “Direct Access,” three administrative officers who had been vetted, trained, and granted a license to use “Direct Access,” and two probation officers.

### Materials

An interview schedule was developed by the first, third, and fourth authors, who have research expertise in information sharing in MTSs, evidence-based policing, and qualitative research and evaluation. Questions were structured to be open ended, in line with an inductive approach and to elicit more in-depth responses. We consulted with a detective sergeant from the IOM team who led the development of “Direct Access” to ensure interview questions were relevant and appropriately worded. The interview schedule included opening questions to explore the role of participants in relation to offender management (e.g., *In what capacity do you interact with probation services [police]?*), followed by questions to understand how “Direct Access” works and how it differs to previous information sharing processes (e.g., Can *you describe the approach of the new information sharing system? How does it differ from what was done before?*), and how “Direct Access” had impacted on information sharing and offender management (*e.g., Can you give me some practical examples of how work processes have changed or developed since the new information sharing system was introduced?*).

### Procedure

After receiving an invitation e-mail and information sheet, police and probation officers who were willing to participate in interviews e-mailed the researchers to arrange a suitable time. They received and completed an electronic consent form prior to participation. Due to the COVID-19 pandemic, all interviews were conducted remotely using Microsoft Teams. Interviews lasted between 20 and 75 min (*M* = 35.42, *SD* = 17.57) and were recorded, transcribed verbatim, anonymized, and then audio files were deleted. Steps were taken during the interview to improve the trustworthiness of the data, including paraphrasing to check researcher interpretation aligned with participant meaning, and asking for concrete examples to sense check (Varpio et al., 2017). Researcher reflections were captured after each interview to inform reflexivity, with notes also being anonymized to maintain participant anonymity.

### Data Analysis

Interview transcripts were analyzed using NVivo^®^ software version 12. A data-driven, inductive thematic analysis approach was adopted to explore police and probation officers’ perceptions and experiences of information sharing difficulties and how the introduction of “Direct Access” impacted this. Thematic analysis is a form of content analysis used to systematically and reliably analyze qualitative data to identify and derive meaning from common themes (Braun and Clarke, 2019). In contrast to other forms of content analysis, thematic analysis focuses on identifying common themes based on content rather than form to derive meaning rather than count frequencies of words or utterances. Thematizing meanings is a key skill required across all forms of qualitative analysis, including grounded theory, conversation analysis, and interpretive phenomenological analysis (Holloway and Todres, 2003). However, thematic analysis differs as it is independent of theory and epistemology, so can be applied across a range of datasets (Braun and Clarke, 2019). Thematic analysis was selected in this study because it allows themes to “emerge from the data” with a level of depth that quantitative research struggles to achieve, but enough flexibility and interpretation to answer the research question (Castleberry and Nolen, 2018).

Data was analyzed in line with Braun and Clarke (2006) six-stage process. This process started with becoming familiar with the content through transcribing interviews. Next, interview transcripts were coded phrase-by-phrase to derive meaning from participants’ responses. As participants were from police and probation backgrounds, *in vivo* coding was considered more appropriate for ensuring that codes were developed using participants’ own voices and reflected their perceptions and experiences (Alhojailan, 2012; Manning, 2017). Data that did not pertain to the research question was not coded, such as participant background details. Initial codes were compiled into similar groups to develop themes. With each interview conducted, codes were revised to ensure that commonalities were identified, and themes produced were of relevance to answering the research question, which is important to the rigor and validity of qualitative research (Castleberry and Nolen, 2018).

All interviews were initially analyzed by the second author and reviewed by the other authors. There is a debate within the qualitative literature regarding use of inter-coder reliability, with some arguing it is beneficial for demonstrating trustworthiness, transparency, and consistency (Connor and Joffe, 2020), whilst others argue the role of a qualitative researchers is not to reveal universally objective truths but to apply their expertise to interpret varied perspectives on an issue (Bauer, 2000). In this study, the second author presented descriptions of themes and the supporting quotes, and the other authors sense checked the validity of themes, scrutinizing whether quotes grouped together had a coherent and logical rationale for how they were organized, and that the theme descriptions provided a meaningful and accurate reflection of the collection of quotes. All authors agreed on the grouping of quotes, but discussions were important for strengthening the clarity and boundaries of theme descriptions to avoid overlap. In addition, participants were e-mailed theme descriptions and anonymized quotes supporting themes to review to ensure they were accurate and representative of participants’ views, to further improve the reliability and robustness of the analysis (Trainor and Graue, 2014). All participants agreed with the themes and evidence supporting them.

## Results

**FIGURE 1:**
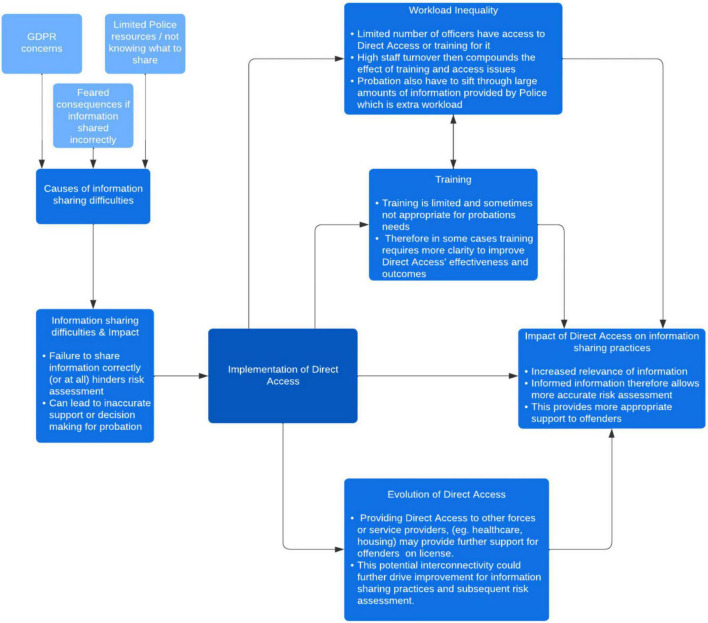
Diagram of how themes relate to one another.

Thematic Analysis of interview transcripts resulted in the following six themes of relevance to understanding the underlying causes of information sharing difficulties in MTSs responsible for offender management, and the impact of “Direct Access” in overcoming them: (i) Information sharing difficulties and impact, (ii) Causes of information sharing difficulties, (iii) Impact of “Direct Access” on information sharing practices, (iv) Workload inequality, (v) Training, and (vi) Evolution of “Direct Access.” These six themes accounted for over 80% of the total quotes. The frequencies of each theme have been calculated to give an overview of the prevalence across interviews, but this should be viewed as contextual information rather than affecting the interpretation of meaning of data (Joffe and Yardley, 2004; see Table 1 for details of the proportion of quotes allocated to each theme and Figure 1 for an overview of how themes relate to one another).

**TABLE 1:** Frequency of themes across interviews.

Theme	Number of participants who mentioned theme		Number of quotes relating to theme
	
	Police	Probation	
Information sharing difficulties and impact	4	7	210 (25.9%)
Causes of information sharing difficulties	4	7	154 (18.9%)
Impact of DA on information sharing practices	3	7	127 (15.6%)
Workload inequality	2	5	102 (12.6%)
Training	3	6	44 (5.4%)
Evolution of DA	3	5	80 (9.8%)

### Information Sharing Difficulties and Impact

Both police and probation officers interviewed discussed the importance of inter-agency information sharing for the effectiveness of offender management but acknowledged that this was both difficult and resource intensive to achieve in practice, requiring *“buy-in from all agencies involved.”* Whilst IOM teams were noted as providing a useful platform for information sharing, this only exists for high-risk offenders, often leaving low-risk or repeat offenders falling into an *“information sharing gap.”*

*“I think the problem is that you’d have to assume there’s something you would need to chase”* – *P9*

“*If they’re not prolific enough to be MAPPA [multi-agency public protection arrangements] or IOM [Integrated Offender Management], they do kind of fall in a gap in the middle, it’s kind of a void”* – *P10*

Information sharing difficulties related to the timeliness, relevance, and efficiency of information sharing. Probation officers noted that prior to the introduction of “Direct Access,” they were reliant on calling the police non-emergency number (101) or police contacts they knew, both of which could incur delays due to busy telephone lines and contacts being on leave. Even when probation officers were able to speak to police, information requests could be rejected, or they could be transferred to different departments as the person they were speaking to did not have the information needed. In addition, relevant information could be withheld because probation officers did not specifically request it as they were unaware of its existence and police did not recognize the need to provide it. For example, police might hold relevant information on arrests or cautions but fail to relay this to probation in a timely manner or at all.

*“You can ring 101 unfortunately you may be on hold for over an hour, if you ring your contact in the IOM [integrated offender management] unit they might be on annual leave and their cover may have no knowledge of the offender you need to look into, so all of the stuff that can go wrong with that. It would often rely on having relevant contacts in relevant agencies – as you said before, that’s getting hold of the right person, at the right time of day, being able to get hold of the right information, however, long it takes to get a call back – it was a bit ad hoc and prone to variation”* – *P11*

*“They normally wouldn’t get any information they’d have to ask for it there a process the police need time to respond to it depending on resources etc. and so there was no real regular information sharing before.” – P2*

*“Some of this information we just wouldn’t have had before, and information sharing is key – we can’t safeguard without the information we gain from these systems*” – *P8*

Delays and failures in police sharing information of relevance to probation officers affected their ability to develop accurate risk assessments and take appropriate safeguarding actions. In the absence of police information and intelligence, probation officers noted being reliant on gut instincts and knowledge gained from meetings with offenders. They felt overly reliant on the honesty of offenders to disclose information, particularly if it related to a breach of license conditions that could result in them being returned to prison.

*“A lot of the stuff we were going on is just gut instinct, in that but you know it [‘Direct Access’] gives us some evidence. You’d have to go off what the offender said, so you’d have two sides of it, and you know the truth is somewhere in the middle” – P3*

*“Our cases won’t necessarily disclose what’s going on, so you could have something that’s kicked off at the weekend and you’d have no idea, they wouldn’t tell you” – P9*

*“It’s down to their honestly really about how everything is – a lot of the time they’re not that honest or I suppose they don’t feel like they’ve been asked the right question to get the right answer.” – P5*

### Causes of Information Sharing Difficulties

Interviewees noted that the reluctance for police to share information and intelligence with other agencies was predominantly caused by issues with GDPR and trust. Interviewees believed there to be a lack of clarity regarding GDPR, which left them open to interpretation. Some police officers viewed GDPR to be restrictive and to create barriers to information sharing due to anxiety over consequences should GDPR be breached. Others viewed GDPR to enable greater information sharing, provided that appropriate boundaries were in place regarding who accesses the data, and how it is used and stored.

*“People are always being encouraged to share information but because of the data protection act and the GDPR as it is now known police organizations have always been reluctant to share information” – P2*

*“Less so with security because we share information with partners in the CJ [criminal justice] all the time, the processes are well understood and actually the legislative landscape around information sharing if you interpret it correctly, it empowers a greater level of information sharing than ever before. It puts appropriate restrictions around it, clearly, but the way I interpret it, I’d rather explain why we have shared than why we haven’t, so that was less of a concern for me.” – P11*

Both police and probation officers also perceived police reluctance to share information to be caused by lack of trust regarding how it would be used, data loss, reputational risk, and consequences of sharing police intelligence with offenders. Probation officers noted that this lack of trust, along with lack of clarity regarding how such information and intelligence could be used and who it could be shared with created anxiety about unintentionally breaching information sharing boundaries and the consequences for doing so.

*“We would often get bounce backs from when we were asking for information like you need to be more specific, we can’t just give you all the information we have on someone*… *It almost felt as if there was a distrust from the police in terms of right what are you going to do with this information are you going to potentially leak something sensitive onto the client” – P3*

*“When you’re audited you need to be giving details of why you’re going into these cases; and you think gosh if I’m doing 45 checks – at the time we didn’t know that’s how many it was going to be – how am I going to remember why I’ve gone into it?” – P5*

### Impact of “Direct Access” on Information Sharing Practices

Both police and probation officers believed the implementation of “Direct Access” required police to take a *“leap of faith”* and place a higher level of trust in probation services. This trust needed to exist at a high level within the organization to sanction the implement of “Direct Access” and associated resources needed to do so. Existing relationships between police and probation services that had developed through IOM teams in the region helped to facilitate this trust. However, the implementation of “Direct Access” also helped to improve trust and relationships because it provided a concrete objective for opening discussions between agencies. The recent murder of Tanis Bhandari by an offender on license in the region and subsequent criticism of information sharing practices lead to the development of “Direct Access” and encouraged a willingness to embrace transformative change and *“dare to share.”*

*“It allows for relationships and helps that co-collaboration on shared issues surrounding risk. Even just the act of bringing Direct Access in, people have had to have conversations and come to agreements that they might not have otherwise had between agencies, so I think that’s a spin off benefit.” – P11*

There was consensus across the probation officers interviewed that implementation of “Direct Access” had improved the timeliness and relevance of information and ability to safeguard effectively. Most probation officers also noted being able to use this information to better challenge offenders on their behavior. Making offenders on license aware that probation officers would know if they had any interactions with police was beneficial for encouraging more meaningful, honest discussion and deterring further offending. Having better access to police intelligence allowed both agencies to identify when offenders on license were struggling so they could provide better welfare support. In addition, having direct access to police records meant that probation officers knew which police officers had responded to incidents involving their client, making it easier to know who to contact if further details were needed about the incident. Probation officers interviewed also felt that the introduction of “Direct Access” created clearer boundaries in terms of who within the probation service could have access to police information, but uncertainty remained regarding expectations about how such information could be used.

*“I think it opens a line of dialog and it encourages them to be open and honest with us, which might seem like a small thing but it’s so useful because our main thing for the officers is assessing risk, and they can only assess risk when they have all of the information”* – *P3*

*“I think it can be an impact on the individual as well, the offender, to know I can’t just leave an area and go somewhere else and commit crime or low-level crime or get involved with something because the probation service is going to end up knowing about it.” – P2*

*“So, I mean even for the other side of it if we have an offender who’s tried to commit suicide, things like that, welfare checks, things like that are on there as well so it’s not just if they’re in trouble”* – *P5*

Both police and probation officers noted that “Direct Access” provided several benefits for improving information sharing and offender management and they would not want to revert to the previous information sharing processes. However, they noted the difficulty of being able to quantify this success because having better access to information and more accurate risk assessments enabled the probation service to take steps to prevention further offending but it is difficult to measure something that has not happened. Nevertheless, all parties agreed that “Direct Access” is a beneficial tool that has the potential to avoid serious further offenses and demonstrates a method for improving multi-agency information sharing within the complex context of offender management.

*“They really are utilizing it and I think we’ve had it for a few years now and I think they would be quite lost without it.” – P8*

*“I think it yeah it definitely helps with safeguarding because the main part of the probation job is around risk management and managing not only the risk to the offender but also the risk to the community at large and if we’re aware of what’s going on with that case, we’re able to put better strategies in place”* – *P3*

*“I think it’s difficult to say how its measured because ultimately this system prevents things happening” – P2*

*“Of 30 reports that we ran, those 60 additional checks were about offending behavior that we needed to address. So that’s 60 activities that we wouldn’t have done, because we wouldn’t have known about them. So in my view, those 60 things could have prevented somebody’s death, someone coming to harm.” – P4*

### Workload Inequality

Whilst police and probation agreed that introducing “Direct Access” had many benefits, there were implementation challenges, most notably in relation to distribution of workload. Police officers noted the level of time investment they made up front in implementing “Direct Access,” including developing data sharing agreements and delivering training for probation services. Police officers believed that by developing “Direct Access” they had fulfilled their obligations for sharing information with the probation service.

“*I think from our perspective from the police our objectives under the SAR [Specified Activity Requirements] have been met and that is to share the information that we have or to let probation access it” – P2*

However, probation officers questioned the equity and fairness of workload distribution as a result of implementing “Direct Access.” Previously, police shouldered the substantial resource burden for sharing information, with demand exceeding capacity, which resulted in delays to responding to information requests from probation. The introduction of “Direct Access” had largely removed this resource burden from police but had shifted it to probation, creating minor tensions between the two services. Police and probation officers acknowledged that both services were overstretched but probation felt the resource burden for information sharing should be more equitable rather than being shouldered by one agency. This disparity was exacerbated because only a small number of probation officers were vetted, trained, and issued with a license to use “Direct Access,” although police had offered to increase this capacity. Additionally, not all probation officers with a license were using “Direct Access,” further increasing burden on an already small group. However, probation officers also noted that, in the short term, the additional workload was a small price to pay for the benefits that “Direct Access” provides.

*“There remains a tension because of that fact, it has gone from being a police resource, to being a probation resource. the police feel like they’ve discharged their duty but in fact they haven’t, and if it looks like resourcing of it is shifted there needs to be some give in terms of the police sharing that resource”* – *P4*

*“The police maybe thought to themselves, we haven’t got the funds and the resources – it would be or it is almost a full time job to do this properly and I would suggest it would be rather than fund an individual job role or a department to take this on, they thought it would be more cost effective sense to them to give us access” – P6*

### Training

Prior to being granted a license for “Direct Access,” probation officers underwent security vetting and training that was delivered by police. This training largely focused on how to use the I.T systems. Whilst these probation officers found the training useful, they questioned the relevance of some of the technical content on system use, which they felt was too in-depth for the day-to-day role of a probation officer. Instead, probation officers wanted more focus directed to preparing them for how to apply “Direct Access” and the greater level of police information and intelligence available to probation. Instead, they had been required to learn on the job and continued to feel anxious about security and what information they could share under “Direct Access.”

*“The training was very police scenario based. So it would be what the police would use Direct Access for, what they would use it for and we were sat there in training thinking well how is this even going to work for us? How does it help?… I suppose it was very new to everybody, we got some handouts and some bits and pieces from that but I think since then it’s been more learning as you go. I think if the training was more about this is what you can tell them, this is what shouldn’t be shared, then that would have been better.” – P5*

Probation officers also struggled to interpret information contained within police systems due to the specialized technical language used by police. Probation officers noted that police would be just as likely to struggle with the technical language and acronyms used by probations. However, they noted that whilst it was beneficial to have timely access to more information relating to offenders on license under their supervision, these language barriers could impact on their ability to interpret and use this information.

*“Over time it’s easier but I must admit when I started it was a bit overwhelming it was like reading another language. if anyone was going to come in and listen to us with our probation acronyms, our new members of staff are always like what are you talking about” – P8*

*“I think sometimes the clarity around or the information they actually put on there is formatted in a way that is for police colleagues to read and sometimes it takes a bit longer for someone who’s not police to go through” – P3*

### Evolution of “Direct Access”

All parties interviewed believed that it would be beneficial to roll out a system like “Direct Access” nationally to support information sharing and coordination of offender management. They also felt that doing so would not only improve information sharing between agencies but also within agencies so that police forces and probation services would have a better system for monitoring offenders on license across geographical areas. In addition, probation officers suggested increasing the wider benefits of a system like “Direct Access” for other agencies that have a role in supporting offenders, including prison service, health care, child support, and housing support, which would create a more collaborative offender management system.

*“Obviously we’re just linked to the one police force down here but if other areas could use this around the country, I think if they could see how we were using it, they’d definitely jump at the chance to have the opportunity as well” – P8*

*“I think there’s this big gap in all forces, probably nationwide, where we could all share this information together and I think they’re doing it with really high-level people but you could do it on the majority, it’s just resourcing it” – P6*

*“it linked in not just probation but then health, other things like housing support, children’s services might find in quite useful.” – P2*

However, police and probation officers also noted some considerations that other regions would benefit from prior to rolling out. These included having structures in place to adapt “Direct Access” over time because no system could stay the same forever and would eventually need to adapt to the changing requirements of agencies. It also included considering the time and resource investment required to set up “Direct Access” and that this would require buy-in from all parties involved. Accordingly, it would be important to be clear about the intended benefits, risks, roles, and responsibilities at the start to secure informed support.

“*If that means they have to adapt or be adjusted over time because the contexts change, systems change or the way we work changes, there’s no such thing as a fixed solution that stands finished and is fixed for 20 years so you know I fully expect we’ll have to adapt the original version of Direct Access”* – *P6*

*“I’ve been fortunate that the people I’ve approached for help assistance and support have been very forthcoming because I’ve explained the benefits. I’ve explained the benefits of doing it and the risks of not doing it, however, in other forces people may need more convincing than that” – P2*

## Discussion

Public inquiries (Bichard, 2004; Pollock, 2013; Plymouth Safeguarding Board, 2020) and academic research (Fitzgibbon, 2008; Goodwin et al., 2012; Ricks et al., 2016) highlight problems with information sharing in the large MTSs responsible for managing offender probation. This can leave probation officers drawing on outdated, unreliable, and incomplete information (Fitzgibbon, 2008; Goodwin et al., 2012; Ricks et al., 2016) that compromises accuracy of risk assessments and decisions regarding what support to provide and whether to recall an offender on license to prison (Florence et al., 2014; Matz and Kim, 2017; Brown, 2018). To date, however, limited research focus has been directed to examining the underlying causes of information sharing difficulties within the complex MTSs responsible for managing offenders on license. Indeed, there has been a lack of research focus directed to examining information sharing practices across other large MTSs with unstable memberships operating in extreme environments with ongoing risk to public safety and shifting information requirements. This poses implications for identifying concrete behaviors and processes that can improve information sharing within these complex, risky environments.

Accordingly, this case study focused on one region of the United Kingdom where a new “Direct Access” initiative was introduced to support information sharing between police and probation services in relation to management of offenders on license. It is the first time a United Kingdom based police organization have permitted an external agency to directly access its I.T. systems, presenting a novel opportunity to examine what works in practice to overcome information sharing challenges in this large and complex MTS, and cross-validate MTS theory to the real-world context of offender management. In line with previous research, underlying causes of difficulties included (i) lack of resources, (ii) difficulties identifying information needs, and (iii) issues with trust regarding how statutory partners will use sensitive information and intelligence. Findings also highlight that whilst allowing statutory partners direct access to I.T. systems can improve the relevance and timeliness of information, *“daring to share”* is not enough to address trust issues without also clarifying expectations regarding information use and perceived workload inequalities. The impact of “Direct Access” on addressing information sharing difficulties is discussed in further detail below.

### Impact of “Direct Access” on Information Sharing Barriers

Both police and probation officers interviewed noted that a key underlying problem affecting information sharing was ability to identify what information to share. This issue is not unique to probation settings – knowing what to share and when is a common problem for large MTSs with unstable membership working toward both interrelated and unique goals (Chatzimichailidou et al., 2015; Waring et al., 2019). Vast amounts of information are distributed across large MTSs, making it increasingly difficult to tailor information to the needs of each party (Stanton et al., 2006; Stanton, 2016; Waring et al., 2018). Information sharing was noted as being more relevant and timelier in multiagency IOMs with stable team membership, but these were resource intensive and only in place for the smaller number of very high-risk offenders on probation. In line with previous findings, both police and probation officers highlighted that unstable membership and changing information requirements made it difficult to develop the familiarity needed to identify what to share, with whom and when in relation to the large numbers of medium- and low-risk offenders released from probation on supervision every day. However, the implementation of “Direct Access” had improved the relevance and accuracy of information sharing. Rather than being reliant on police to correctly identify what information was relevant to share and when, or on availability of police resources to address information requests, “Direct Access” allowed probation to access information directly when they needed it. In this respect, “Direct Access” was noted as a beneficial tool for improving the relevance and timeliness of information access within this large and complex MTS.

In line with previous research (Kim et al., 2016; Matz and Kim, 2017), another key barrier to information sharing was limited resourcing. Police and probation officers noted that with continued funding cuts, they were being expected to do more with less. Consequently, limited resources were being prioritized to address internal issues rather than partnership working. Evidence from the MTS literature suggests that this prioritization of intra-agency over inter-agency goals is exacerbated by disparities in membership, whereby one group is viewed as receiving greater prioritization or benefit over others (Shuffler et al., 2015). Similar issues have been identified in offender management settings (Kim et al., 2016), and current findings further support this. Police and probation officers interviewed recognized that both parties were in a difficult position as information demands often exceeded resources. Having to respond to multiple requests from probation for information relating to many different offenders on license was placing large demands on police resourcing, and they were not always able to respond to these requests due to other intra-agency competing demands. For probation, there were also resource implications in terms of time spent making repeated calls to various police officers or units trying to find someone to provide the information needed, sometimes without success. Probation also perceived police to be prioritizing their own internal goals over sharing information to address inter-agency goals.

Whilst “Direct Access” was introduced to reduce these resourcing demands as well as improving information sharing, findings were mixed. Both police and probation officers believe that “Direct Access” had reduced resourcing demands for police by pushing responsibility to probation services. Previous research indicated that information sharing was viewed less favorably by police than probation due to the belief that it placed greater resource demands on police, but probation received the greater benefit (Kim et al., 2016). Such disparities in MTS membership and perceived imbalance between workload and benefits can affect willingness to invest in inter-agency working (Shuffler et al., 2015). Findings from the current study highlighted that for police, the reduction in resourcing demands achieved by implementing “Direct Access” was appropriate. However, probation officers believed police had passed these resourcing demands to probation and that this was not a fair balance of workload between services. Whilst the introduction of “Direct Access” led to improved information access and risk assessment for probation services, it also increased resourcing demands and shifted the perception of workload imbalance from police to probation.

The final information sharing barrier noted by both police and probation services was lack of trust over how information would be used and who it would be shared with, in line with previous research highlighting a culture of secrecy within policing (Kelley, 2003; Maras, 2017; Gil-Garcia et al., 2019). This is often underpinned by concern that investigations will be compromised by offenders being made aware of police activities (Maras, 2017; Aden, 2018). Feedback from both probation and police officers paralleled these previous findings, but both parties also noted that lack of clarity regarding the Data Protection Act and GDPR exacerbated this, creating misconceptions about what information could be shared with boundaries in place. Both police and probation officers felt that introducing “Direct Access” demonstrated a meaningful shift in police overcoming cultural barriers and “*daring to share*.” Previous research highlights that these cultural barriers can be hard to change (Maras, 2017). Police and probation officers highlighted the key driver to this change had been the murder of Tanis Bhandari by an offender out on license, creating a clear shared goal between police and probation to take action to improve information sharing in offender management. However, whilst “Direct Access” was a step in the right direction, probation still perceived there to be some issues with trust. This stemmed from concern about the lack of clarity provided by police during training about expectations for who information could be shared with and how it could be used, creating anxiety about potential consequences if they were found to have misused information. Previous MTS research highlights the importance of having a clear shared goal and understanding of one another’s roles and responsibilities for avoiding conflict, gaps, or duplications (Davison et al., 2012; Shuffler et al., 2015; Waring et al., 2018, 2019).

## Limitations and Future Research

Whereas most offender management research focuses on the perspectives of a single agency (Kim et al., 2010), the current study drew on interviews conducted with both police and probation, which was beneficial for comparing perspectives and experiences. We also took several steps to address criticisms often leveled at the reliability and robustness of qualitative research (Noble and Smith, 2015), including interview schedules being developed by academics and practitioners with expertise in MTS information sharing and offender management, paraphrasing during interviews to sense check understanding with participants, transcribing interviews verbatim, and sharing themes with participants to sense check analysis and interpretation (Tracy, 2010; Levitt et al., 2016).

However, the study is not without limitations. To date, “Direct Access” has only been implemented in one region of the United Kingdom and its implementation was driven by the recent murder committed by an offender on license. It is difficult to determine the extent to which these findings can be generalized to other regions that have not experienced this type of galvanizing event. Although findings parallel those found in other large MTSs operating in different extreme environments, such as disaster response (Waring et al., 2018, 2019), suggesting commonalities in underlying causes of information sharing problems, further research is needed to examine the extent to which tools like “Direct Access” may be beneficial for improving information sharing in other contexts. It should also be noted that information sharing with police is just one aspect of probation services multidimensional approach to responding to offenders. Future research is needed to explore other strategies probation have in preventing crime, such as increasing understanding of offenders and building positive trustful relationships. However, feedback from probation officers suggested that “Direct Access” had a role to play in facilitating relationships with offenders through opening conversations using the wider information available through this system to challenge offenders and to offer better tailored support.

## Conclusion and Implications

Overall, findings from the current study highlight three key causes of information sharing difficulties between police and probation services, (i) not knowing what information to request or share, (ii) limited resources, and (iii) lack of clarity about GDPR, and concern about the consequences of breaching this. These barriers can result in delays and failures to share relevant information, which hinder accuracy of risk assessments and ability to safeguard. The implementation of “Direct Access” has increased the relevance and timeliness of police information available to probation, improving risk assessment and ability to appropriately support offenders on license. However, the extent to which “Direct Access” can achieve these positive outcomes is affected by effectiveness of training, and this requires greater clarity on how “Direct Access” and the information accessed can be used and the conditions under which it can be shared, including clearer guidance for both police and probation on Data Protection and GDPR. In addition, the success of “Direct Access” is also impacted by distribution of workload, with probation viewing this to be pushed back onto their service with only a limited number of probation officers trained and with a license to use “Direct Access.” Implementation of new information sharing processes requires greater focus on clarifying workload distribution and ensuring appropriate numbers of staff are in place to undertake key information sharing roles within large MTSs. Feedback from police and probation services suggests that expanding the use of “Direct Access” to other regions of a country and other services with involvement in the management of offenders on license (including health, housing, and social care) has the potential to further improve information sharing practices, risk assessment, and safeguarding.

## Data Availability Statement

The raw data for this study is the property of Devon and Cornwall Police and will not be made publicly available. Requests to access the datasets should be directed to dataprotection@devonandcornwall.pnn.police.uk.

## Ethics Statement

The studies involving human participants were reviewed and approved by Health and Life Sciences Research Ethics Committee (Psychology, Health and Society), University of Liverpool. The patients/participants provided their written informed consent to participate in this study.

## Author Contributions

SW, SG, and LA designed the study, including materials and gained ethical permission. ET carried out interviews and undertook qualitative analysis under the supervision of LA and SW. SG trained ET in interview techniques. SW wrote the introduction and discussion sections. All authors reviewed analysis and contributed to writing the method and results sections.

## Conflict of Interest

The authors declare that the research was conducted in the absence of any commercial or financial relationships that could be construed as a potential conflict of interest.

## Publisher’s Note

All claims expressed in this article are solely those of the authors and do not necessarily represent those of their affiliated organizations, or those of the publisher, the editors and the reviewers. Any product that may be evaluated in this article, or claim that may be made by its manufacturer, is not guaranteed or endorsed by the publisher.

## Footnotes

1
www.gov.uk
